# Magnesium Filled Polylactic Acid (PLA) Material for Filament Based 3D Printing

**DOI:** 10.3390/ma12050719

**Published:** 2019-03-01

**Authors:** Iulian Antoniac, Diana Popescu, Aurelian Zapciu, Aurora Antoniac, Florin Miculescu, Horatiu Moldovan

**Affiliations:** 1 Physical Metallurgy, Department of Metallic Materials Science, University Politehnica of Bucharest, 060042 Bucharest, Romania; antoniac.iulian@gmail.com (I.A.); antoniac.aurora@gmail.com (A.A.); f_miculescu@yahoo.com (F.M.); 2 Department of Machine and Manufacturing Systems, University Politehnica of Bucharest, 060042 Bucharest, Romania; aurelianzapciu@yahoo.com; 3 Faculty of Medicine, University Titu Maiorescu of Bucharest, 040441 Bucharest, Romania; h_moldovan@hotmail.com

**Keywords:** magnesium filled PLA, vitamin E, filament feedstock, material extrusion, 3D printing, microstructure characterization

## Abstract

The main objective of this research is to prove the viability of obtaining magnesium (Mg) filled polylactic acid (PLA) biocomposites as filament feedstock for material extrusion-based additive manufacturing (AM). These materials can be used for medical applications, thus benefiting of all the advantages offered by AM technology in terms of design freedom and product customization. Filaments were produced from two PLA + magnesium + vitamin E (α-tocopherol) compositions and then used for manufacturing test samples and ACL (anterior cruciate ligament) screws on a low-cost 3D printer. Filaments and implant screws were characterized using SEM (scanning electron microscopy), FTIR (fourier transform infrared spectrometry), and DSC (differential scanning calorimetry) analysis. Although the filament manufacturing process could not ensure a uniform distribution of Mg particles within the PLA matrix, a good integration was noticed, probably due to the use of vitamin E as a precursor. The results also show that the composite biomaterials can ensure and maintain implant screws structural integrity during the additive manufacturing process.

## 1. Introduction

Additive manufacturing technology (also known as Rapid prototyping or 3D printing) has been on the market since 1988—the year of the launch of the first machine (SLA-1) based on stereolithography (SL) processes. However, this field developed greatly only after the patent for fused deposition modeling (FDM) expired in 2009. Nowadays, this process is being known under many designations, such as FFF (fused filament fabrication), 3D printing or under the standardized name of material extrusion (ME). The so-called “3D printing democratization” brought many improvements in the hardware and software of AM machines, as well as in the development of a versatile range of build materials (feedstock used in AM).

Numerous applications for the medical field have been developed using AM technology, such as anatomical mockups for surgery training, planning or simulation [1,2], personalized surgical guides [3,4], tissue engineering scaffolds [5,6], customized implants [7,8], etc. Not all these applications are based on FFF, which usually uses thermoplastics, such as ABS (acrylonitrile butadiene styrene), PC (polycarbonate), PLA (polylactic acid), PETG (polyethylene terephthalate glycol), Nylon PA (polyamide), PEEK (poly-ether-ether-ketone) [9], or their composites. For instance, metal alloy implants have been manufactured using selective laser melting (SLM) or electron beam melting (EBM), while for anatomical mockups, PolyJet and SL processes have been preferred. However, the advantages offered by the extrusion-based process, such as low cost of machines and build materials or easy setting of process parameters depending on feedstock type, have determined the current popularity and widespread adoption of AM-based ME processes. Therefore, for the application of interest in this study, to develop innovative biocomposites and further use them to obtain implants, FFF represented a practical and straightforward manufacturing solution.

Literature presents several types of research on developing new composites for FFF, using two main approaches:

- Producing feedstock filament by mixing matrix and filler: ABS and glass [10], polyamide and short carbon fiber [11], ABS and nano-fiber [12], PLA and metal [13], etc.
- Inserting continuous carbon fibers into the matrix (PLA or ABS) or inserting continuous natural fibers in different polymers inside the extrusion nozzle by “in-nozzle impregnation” [14,15,16].

The main objective of the studies on these topics has been to characterize the material’s structure and establish correlations between microstructural characteristics, processing parameters, and their mechanical properties (usually tensile strength, Young’s modulus, and yield strength). Aspects like the influence of fiber orientation over the mechanical properties, as well as the reinforcement effect over specimen porosity, have also been studied. Typically, the results have shown superior mechanical properties of specimens made with polymeric-based composites in comparison with the similar specimens made just with polymer material [14]. Samples manufactured using various process parameters have been mechanically tested, and their microstructure has been characterized using various techniques [17,18]. Comparison with injection molded parts has been made in order to evaluate the suitability of the new filaments for producing functional objects [19].

Literature in the field of producing biofilaments for material extrusion-based AM processes is scarce. Gkartzou et al. have presented the use of a bio-thermoplastic material made with PLA and lignin biopolymer in the production process [20]. Different lignin concentrations have been tested, 5 wt.% content being considered optimal for manufacturing 1.75 mm diameter filament for FFF. PLA and lignin have been melt, mixed using a Brabender internal mixer, and then have been cast into a mold for fabricating the specimens for characterization. 

Zhao [9] and Vaezi [21] have manufactured PEEK parts for medical applications using the ME process. In order to obtain defect-free parts without delamination or warpage, different process settings have been tested [21]. Also, mechanical strength and in vitro cytotoxicity tests have been performed in some studies [9]. 

No references were found on the usage of PLA-Mg (magnesium) composites as filament feedstock for FFF and for medical applications; thus this paper fills in that niche. In the current research, filaments for FFF were produced from two PLA + Mg + vitamin E (α-tocopherol) compositions and then used for manufacturing test samples and ACL (anterior cruciate ligament) screws on a low-cost 3D printer.

## 2. Materials and Methods 

In FFF, objects are manufactured by melting and extruding thermoplastic material through a nozzle and depositing it as a layer. Nozzle trajectory is generated based on sections obtained by slicing a digital three-dimensional model of the object. First, roads of material are deposited and form layers, then layers are superposed and adhere to each other forming the object. A layer is typically built by depositing perimeters and then filling in the remaining space with roads of material using different strategies and filling patterns. 

In this study, FFF was used to obtain ACL interference screws based on filaments prepared from PLA-Mg pellets using a custom extruder.

**Figure 1:**
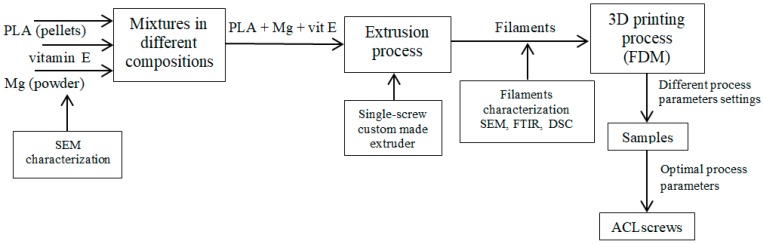
The process of manufacturing implant screws from Mg filled PLA composite.

The following steps had been carried out during the research (Figure 1): prepared two compositions of Mg filled PLA using vitamin E as precursor → used the compositions to obtain filaments for FFF process → 3D print test specimens with different manufacturing settings (extrusion temperature, bed temperature) for identifying suitable process parameter values → 3D print implant screws.

### 2.1. Obtaining Raw Materials

**Figure 2:**
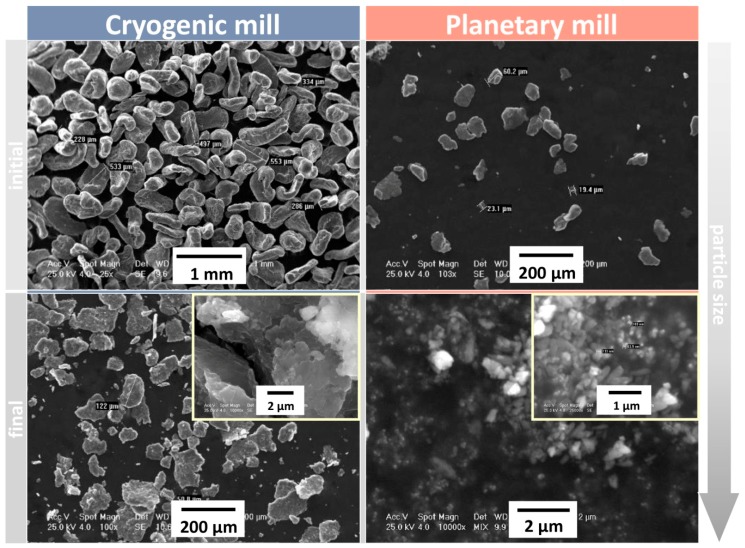
SEM analysis for magnesium powder along different steps of grinding.

A cryogenic mill (Cryomill) and a planetary ball mill (PM 400, Retsch, Haan, Germany) were used for performing Mg particles grinding steps (in cycles of 48 h each). After successive grindings, Mg particles with initial dimensions of approximately 500 µm were reduced to particles smaller than 125 µm (Figure 2).

PLA material (natural, without color additives) from the same producer (Formfutura BV, Nijmegen, The Netherlands) was acquired as granules (pellets) and as 1.75 mm diameter filament.

Two compositions of PLA-Mg were prepared for obtaining filaments using a custom made single-screw extruder:

- 150 g of PLA cylindrical pellets obtained from cutting 1.75 mm diameter filament in 4–6 mm length pieces were mixed with 6 g of Mg 100 µm particles—Composition 1;
- 150 g of PLA spherical pellets of 5 mm diameter were mixed with 4 g of Mg 125 µm particles—Composition 2.

For ensuring Mg powder adherence to the PLA pellets, 2 g of liquid vitamin E was used in both compositions. First, PLA pellets were mixed with vitamin E, and then Mg particles were added to the compositions. Mg adhered to the surface of the PLA pellets, but during the mixture and melting process in the extruder, Mg particles integrated within the PLA material. Thus, the method proposed for obtaining filaments could not ensure a controlled distribution of Mg particles within the PLA matrix but could ensure good material integration as proved by SEM analysis.

### 2.2. Obtaining PLA-Mg Filaments

Biocomposite filaments with diameter 1.75 mm ± 0.11 mm were produced for use as feedstock for an Anet A8 low-cost 3D printer (Shenzhen Anet Technology Co., Ltd., Shenzhen, China) with a 0.4 mm diameter nozzle. The biocomposite filaments in the new compositions were first used to manufacture cylindrical test specimens of 18 mm diameter and 5 mm height, with different process parameters settings. Specimens were manufactured in approximately 7 min each, using 100% infill, 2 perimeters and a ±45° raster layer deposition strategy at a print speed of 50 mm/s. The layer width was 0.2 mm for all specimens, and no support structures were required. These specimens were 3D-printed by varying the extrusion temperature (180 °C, 190 °C, 200 °C) and bed temperature (40 °C, 50 °C) in order to find the suitable process settings for the material in terms of layer deposition quality and road adhesion. Both prepared compositions were used to manufacture specimens.

For obtaining the experimental filaments, a classic extruder was built. The PLA-Mg-vitamin E composition stored in a container was gravitationally fed into the extruder. A steel auger screw actuated by an electric motor pushed the pellets through a steel pipe into a melt chamber situated at the end of the pipe, the molten material was then forced through a brass nozzle. The melting chamber and the brass nozzle were heated using a 220 VAC, 200 W ceramic heater (LJXH, Shenzhen, China). Upon exiting the nozzle, the filament was rapidly air cooled using fans. A K-type thermocouple with a precision of ±1.50 °C monitored the extrusion temperature. 

Initially, a nozzle with a 1.5 mm diameter for obtaining filament of approximately 1.75 mm diameter was used. However, this nozzle diameter produced a filament with less than 1.2 mm diameter, unsuitable for the Anet A8 3D printer (Shenzhen Anet Technology Co., Ltd., Shenzhen, China). Then, the 1.5 mm diameter nozzle was replaced with one with 2 mm diameter. 

At this stage of the research, different filaments were produced by varying the rotation speed of the auger screw. It was noticed that a higher speed determined the filament to adhere to the nozzle at the exit. Several extrusion temperatures were used for obtaining filaments: 170 °C, 175 °C, 180 °C, 190 °C, and 200 °C. Two fans were used to cool the filament. The optimal extrusion temperature for obtaining filaments was 170 °C for both cylindrical pellets and spherical pellets. Other tested temperatures had resulted in a too soft (molten) filament at the exit from the nozzle or too brittle after cooling.

In the filament production stage, a material composition containing vitamin E was gradually heated from room temperature to 170 °C as it moved inside the extruder. The time spent by the material inside the melt zone was less than 2 min. During 3D printing, filament including vitamin E was heated from room temperature to extrusion temperatures of up to 200 °C for about 5 min. Based on the results presented in [22], vitamin E deterioration at these temperatures was relatively reduced if the exposure time was short as was the case in this study. However, even if the temperature would degrade the vitamin E content partially or totally, its desired effect of ensuring Mg particles adherence to the PLA matrix was achieved.

### 2.3. Filament Characterization

**Figure 3:**
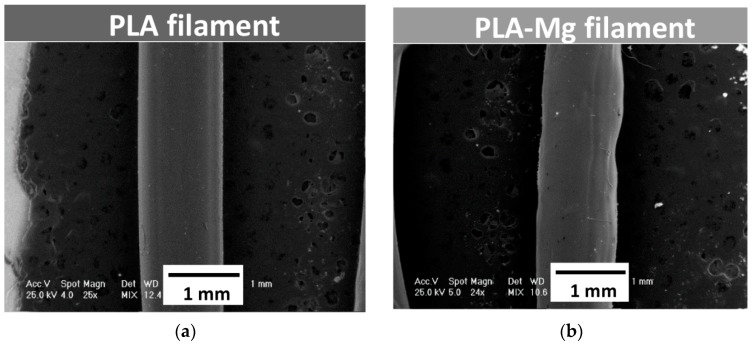
SEM analysis of the experimental filament-general view: (**a**) PLA; (**b**) PLA-Mg in Composition 1 showing the non-homogeneities in diameter due to the presence of Mg particles.

The PLA filament and the two newly produced PLA-Mg filaments were investigated using the Phillips XL-30 ESEM (FEI/Phillips, Hillsboro, OR, USA) scanning electron microscopy device coupled with EDS (EDAX Sapphire equipment, Mahwah, NJ, USA). The results are shown in Figure 3.

**Figure 4:**
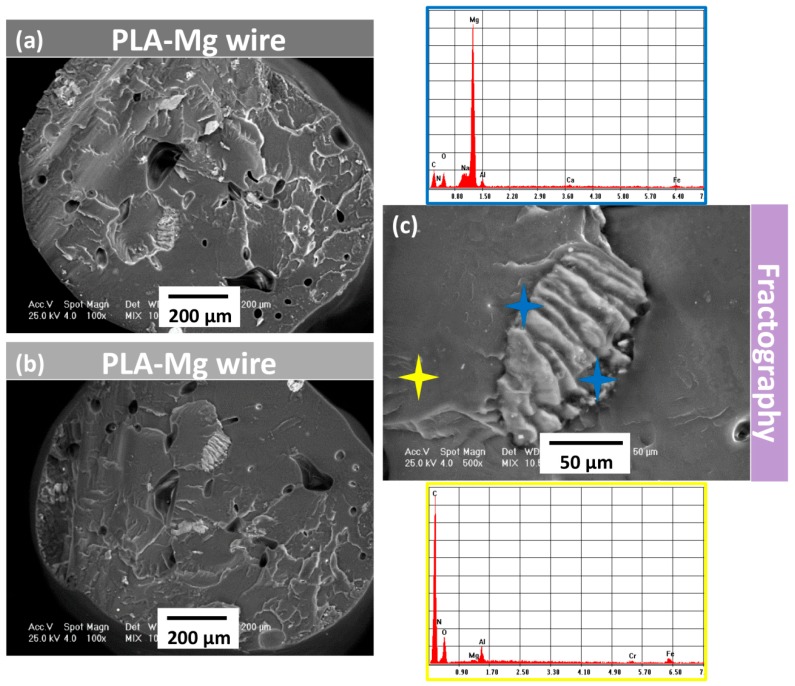
SEM analysis of the experimental wires-fracture section: (**a**) PLA-Mg wire in Composition 2, (**b**) PLA-Mg wire in Composition 1; (**c**) SEM detail in the fracture section coupled with EDS analysis show the presence of Mg particles.

Also, in order to evaluate the integration of Mg particles in the PLA matrix, SEM analysis was performed on filaments after their fracture (Figure 4). SEM analysis was also carried out on the fractures surfaces to see if filaments defects, such as air inclusions, were present. The images were collected at an accelerated voltage of 25 kV.

**Figure 5:**
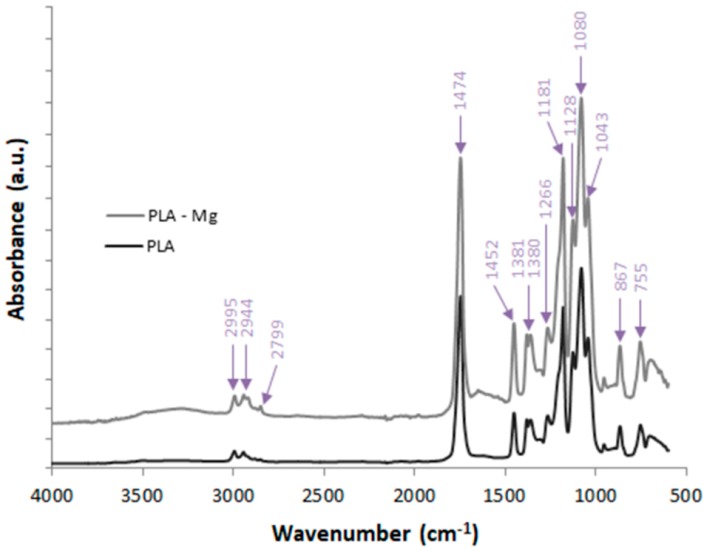
FTIR investigations: PLA, PLA-Mg (Composition 1).

FTIR investigations were also performed on PLA filament and PLA-Mg filaments using an FT-IR JASCO 6200 spectrometer (JASCO, Easton, MD, USA). The results are shown in Figure 5.

**Figure 6:**
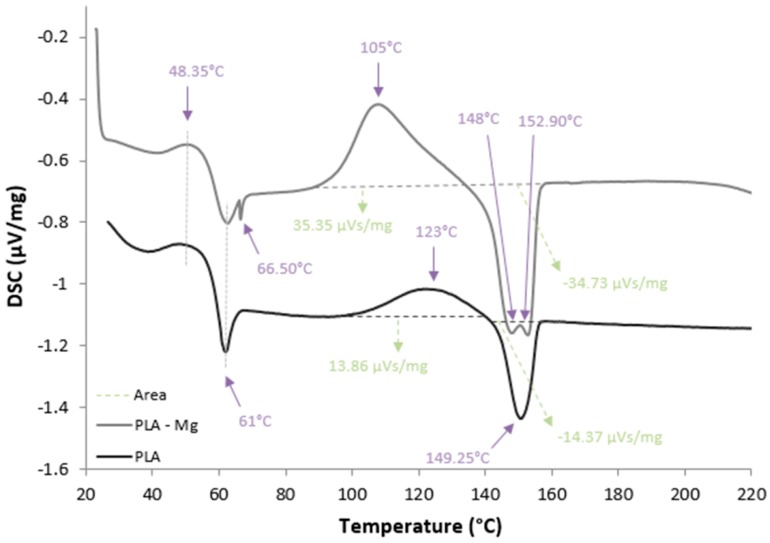
DSC curves of the experimental samples: PLA sample, PLA-Mg (Composition 1).

The differential scanning calorimetry (DSC) tests were done with the use of a DSC 204F1 Phoenix manufactured by Netzsch (Selb, Germany). The analyses were performed in He protective gas with 99.999% purity. The heating rate of specimens with an average weight of 6 mg was 10 °C/min. The DSC curves are shown in Figure 6.

Even if the entire mass of the filler particles comprised sub-micrometer size particles, the sieving particle sorts are less than 100 μm (see Figure 4). This maximum value is less than 10% of the filament diameter, which ensures a small particle agglomeration probability. During experimental process development, the filament extrusion process revealed a variety of cross-section shapes and discontinuities. In order to improve the homogeneity and particle distribution within the volume of the polymer, additional oily vitamin E was used. This contributed to the lubrication of individual Mg powder particles, resulting in limiting agglomeration. Normally, Mg powder reacts with oxygen in the atmosphere and using vitamin E helps to inhibit this process.

## 3. Results

### 3.1. Filament Characterization

Figure 3 shows the results of SEM analysis performed on PLA filament (Figure 3a) and PLA-Mg filament in Composition 1, as an example, with 1.5 mm diameter extrusion nozzle (Figure 3b). No microstructure differences were noticed between filaments in Compositions 1 and 2.

It was observed that the diameter of the filament containing Mg was not constant—it varied between 1.11 mm and 1.17 mm. Some irregularities were observed at the surface of the experimental filaments made from PLA-Mg. As already mentioned, changing the extruder nozzle to 2 mm diameter determined to obtain filaments of around 1.75 mm diameter. Also, due to an inconstant filament diameter, tuning the filament extruder was necessary. This was performed by establishing a better correlation between extruder screw speed and the speed of the spool of which filament is placed at the exit from the nozzle.

In order to analyze the integration of the Mg particle in the PLA matrix, SEM analysis was also performed on the filaments after their fracture. The fracture pattern was similar for both experimental filaments being characteristics for polymers like PLA. According to SEM analysis in the fracture area (Figure 4), good integration of Mg particles, probably due to the use of vitamin E as a precursor, could be observed. 

On the other hand, the distribution of Mg particles could not be controlled in the filament preparation stage; thus Mg particles are distributed heterogeneously within the filament.

FTIR investigations were also performed (Figure 5) on PLA filament and PLA-Mg filaments showing that no new functional groups combinations appeared when filling PLA with Mg:

- Between 3200 and 2800 cm^−1^—bands due to the symmetrical and asymmetrical stretching vibration of the CH group;
- Between 1500 and 1000 cm^−1^—bands assigned to the asymmetrical and symmetrical stretching vibration of the C–O–C;
- Between 960–830 cm^−1^ and 1430–1520 cm^−1^—bands assigned to the stretching vibration and bending vibrations, respectively, of the ${\mathrm{CH}}_{3}$ group;
- At around 1748 cm^−1^ appear bands due to the stretching vibration of the C=O group.

Experimental results obtained after DSC investigations of samples (PLA, PLA-Mg) are shown in Figure 6.

According to the DSC curves, the presence of Mg filler induces the reduction of the glass transition temperature. The data were interpreted by assuming that interactions between filler particles and the polymer matrix reduce molecular mobility and flexibility of the dimer chains in the vicinity of the filler.

### 3.2. Implant Screws Characterization

**Figure 7:**
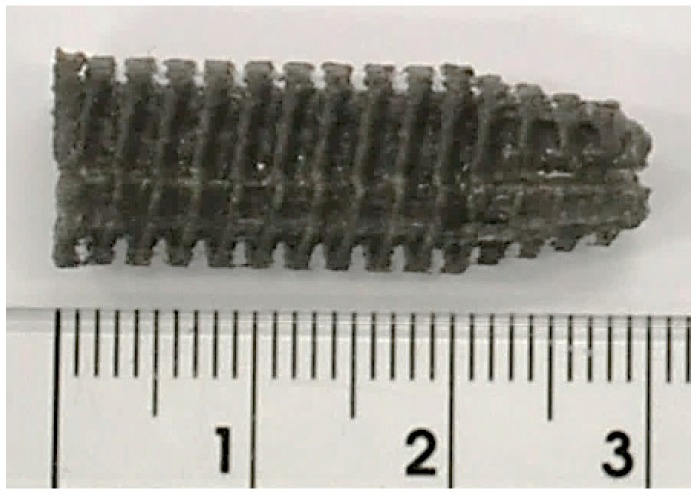
ACL screw from PLA-Mg-vitamin E (Composition 1).

3D-printed ACL screws in the two PLA-Mg compositions (Figure 7) were manufactured for investigating if filaments are suitable for building fine geometrical features and if they can ensure screws structural integrity during the building process. The implants screws were manufactured at 200 °C extrusion temperature and 50 °C bed temperature, these values were being considered optimal after analyzing the specimens obtained by varying process parameters.

**Figure 8:**
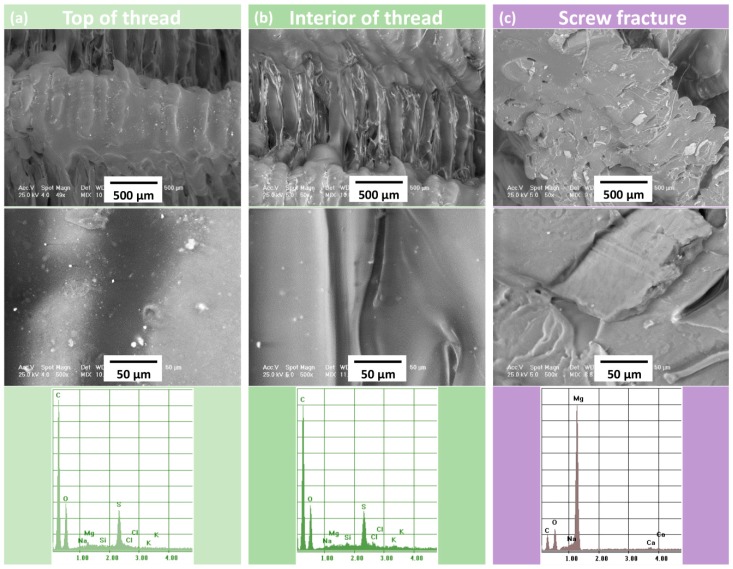
Representative results of SEM and EDS analysis: (**a**) analysis on the top of screw, (**b**) analysis of the Mg particle embedded in PLA matrix, (**c**) transverse screw fracture after immersion in liquid nitrogen.

Comparative aspects obtained through SEM and EDS analysis regarding the morphology and composition of the 3D printed implant in different areas and conditions are presented in Figure 8. The product at low magnification was emphasized separately, in order to indicate macroscopic structures. At higher magnifications, details regarding the Mg particles embedding with smooth interfaces were revealed. No gaps were found between Mg particles and polymer mass.

Figure 8 shows also the EDS spectra corresponding to the analysis of the total surface of the thread crest. The particle-free polymer and the visible Mg particle, respectively, are shown in Figure 8b.

According to the images presented in Figure 8, one can observe a good adhesion between the successive layers deposited by printing, the differences being strictly morphological. On the screw thread heights, the melting was achieved, the result being noticed through the reduction of surface roughness in those areas and by the clear interconnection of the composite fibers. 

Between screw threads, the morphology indicates a higher roughness, with less rounded shapes compared to peak areas (Figure 8c).

The fractographic analysis performed in liquid nitrogen indicated a very good integration of Mg particles into the PLA matrix. This was probably determined by vitamin E treatment before addition of Mg. The Mg particles were completely embedded in the polymer mass, the dispersion being uniform. No particle agglomerations were observed, and they did not influence the adhesion of the deposited filaments and surface morphology.

## 4. Discussion

The capability of magnesium-based materials to degrade into human environment recommends them for a multitude of medical applications. In contrast to traditional metals used as biocompatible materials for which development aims are to improve the mechanical properties, corrosion resistance, and production costs, for biodegradable magnesium, the aim is the formation of an active interface between biodegradable Mg particles and surrounding biological environment in vitro and in vivo.

Various studies have focused on the biological environment-biomaterial interaction and cellular mechanisms to highlight how the materials are biologically influenced by the dissolution of corrosion by-products from the bulk of the material [23,24]. Magnesium has also been tested by many research groups in order to develop various medical applications, especially after it was established that implanting Mg devices shows no significant changes in blood composition [25,26,27,28]. 

Some studies have also explored how polycaprolactone (PCL) and PLA polymer coatings influence the corrosion behavior and drug-eluting kinetics for biodegradable stent applications made from Mg alloys. PLA is a biodegradable polymer which is characterized by good mechanical properties and high biocompatibility. Moreover, the end products can be removed by the body through fluids or metabolized by the liver and kidneys [29,30]. Xu and Yamamoto [31] have prepared biodegradable polymer films of PLA and PCL on Mg by spin coating in order to improve its early corrosion resistance and biocompatibility. Their results have shown that PLA has better adhesion strength with Mg substrates than PCL and that cells are more proliferative on PLA despite the fact that the amorphous PLA and semi-crystalline PCL coatings display uniform and nonporous surface structure on Mg. The results of in vitro dynamic degradation of pure Mg with PLA and PCL coatings have also shown that PCL has a better corrosion resistance in modified simulated body fluid solution than PLA. Also, experimental research performed by Gollwitzer [32] showed that a PLA coating for orthopedic implants based on three different Mg-based alloys had good stability.

Despite the interest in developing new materials for ME, there are no studies on obtaining Mg filled PLA materials and using them as filaments for this process, thus benefiting of the advantages of AM technology in terms of design freedom and fast customization.

During the initial phase of the experiments, it has been noticed that Mg powder adheres to the feeding container walls much more than to the pellets. Therefore, a solution was needed for making the Mg particles stick to PLA during filament fabrication. Liquid vitamin E, a natural biological agent, was chosen for the lubrication of Mg particles and for reducing the probability that Mg particle react with the oxygen rich environment. 

Vitamin E interaction with the human body is widely discussed in the literature. The well-known application of the incorporation of vitamin E in polymeric materials is its use for doping UHMWPE (Ultra-high-molecular-weight polyethylene). Vitamin E has been used to dope UHMWPE in order to provide oxidation resistance upon sterilization, without affecting UHMWPE’s mechanical properties. Galliera et al. showed that in bone cells, the vitamin E-blended UHMWPE induced an osteoimmunological response that had a positive effect on the osteolysis induced by wear debris [33].

Kyomoto et al. have demonstrated the fabrication of highly hydrophilic and lubricious poly(2-methacryloyloxyethyl phosphorylcholine) (PMPC) grafting layer onto the antioxidant vitamin E-blended CLPE (HD-CLPE(VE)) surface, showing that the PMPC-grafted HD-CLPE(VE) provide simultaneously high-wear resistance, oxidative stability, and mechanical properties [34]. The incorporation of the antioxidant vitamin E (α-tocopherol) has been proposed as an alternative to post-irradiation melting treatment after gamma-ray irradiation to avoid oxidation by Bracco et al. [35]. Most frequently, vitamin E is incorporated in polyethylene (PE) by blending it into PE powder before consolidation to form a molded bar or sheet stock, and subsequent gamma-ray irradiation is carried out for cross-linking. In this process, the vitamin E concentration and irradiation dose are relatively easy to control and can be optimized [34,36]. The presence of vitamin E in the substrate prevents degradation caused by oxidation, but it can also reduce the efficiency of cross-linking and PMPC grafting while the vitamin E itself reacted [36].

Kyomoto has annealed vitamin E-blended polyethylene at 120 °C for 7.5 h and has mentioned that it is thought that the stabilization of the residual free radicals with an antioxidant, such as vitamin E, was necessary as an additional or alternative process [34]. Moreover, other studies have reported that vitamin E and wear particles containing vitamin E can possibly reduce the inflammatory cellular responses [37,38]. 

Reno et al. have suggested that it could act as a stimulating factor for osteoblast proliferation and maturation in the case of vitamin E addition to poly(D,L)-lactic acid [39]. Another study, performed by Reno, has indicated that vitamin E addition improved P(D,L)LA haemocompatibility and vitamin E presence caused an increase in polymer surface wettability and human plasma protein adsorption [40].

Also, according to the study performed by Misra et al. [41], incorporating vitamin E as an additive in composites had the ability to engineer the surface of the composites by promoting higher protein adsorption and increasing the hydrophilicity.

In a recent study published by Salawi et al. [42], it has been mentioned that some specific polymer/vitamin E composites could be used in the future development of industrial, dental, and wound healing applications, as adhesives and traps in industrial products, as extruding agents, and potential carriers for drugs for transdermal drug delivery applications.

## 5. Conclusions

The research presented in this paper investigated the development process of new composite material, Mg filled PLA in the form of filaments, proving the suitability of this composite for AM-based on material extrusion. To the best of our knowledge, this topic was not considered in the literature. ACL screws were manufactured using these filaments, the results showing that the new composite biomaterials can ensure and maintain the structural integrity of implant screws during manufacturing. Material characterization and research on ME process parameter settings are also presented in this paper. 

An important aspect revealed by the research is related to the use of vitamin E for assuring Mg particles adherence to the PLA pellets during the raw materials preparation phase. 

Regarding filament production stage, the experiments performed show the need to optimize the process for ensuring a constant diameter and for avoiding the formation of air bubbles that negatively affect the characteristics of filaments and 3D-printed specimens and implants. 170 °C extrusion temperature and a 2 mm extrusion nozzle were determined as optimal for obtaining a 3D printing filament of about 1.75 mm diameter.

In the 3D printing process, temperatures for the nozzle and build platform (print bed) were investigated for determining the values that can provide good adhesion between roads and layers, as well as a good adhesion of the print to the platform. The following settings were considered optimal: printing temperature: 200 °C, build platform temperature 50 °C. Other manufacturing parameters were: nozzle diameter 0.4 mm, layer width 0.2 mm, ±45° raster, one perimeter, print speed 50 mm/s, infill density 100%.

Further research will be focused on filament composition optimization in accordance with the results of mechanical tests and biodegradation studies. Also, the strategy of layer filling will be varied for analyzing the influence of this parameter over the mechanical properties of samples. 

## References

[1] O’Brien E.K., Wayne D.B., Barsness K.A., McGaghie W.C., Barsuk J.H. (2016). Use of 3D Printing for Medical Education Models in Transplantation Medicine: A Critical Review. Curr. Transplant. Rep..

[2] Hochman J.B., Kraut J., Kazmerik K., Unger B.J. (2014). Generation of a 3D printed temporal bone model with internal fidelity and validation of the mechanical construct. Otolaryngol. Head Neck Surg..

[3] Popescu D., Laptoiu D. (2016). Rapid prototyping for patient-specific surgical orthopaedics guides: A systematic literature review. Proc. Inst. Mech. Eng. H.

[4] Popescu D., Laptoiu D., Marinescu R., Botezatu I. (2018). Design and 3D printing customized guides for orthopaedic surgery—Lessons learned, Rapid Prototyping Journal. Rapid Prototyp. J..

[5] Lara-Padilla H., Mendoza-Buenrostro C., Cardenas D., Rodriguez-Garcia A., Rodriguez C.A. (2017). Influence of Controlled Cooling in Bimodal Scaffold Fabrication Using Polymers with Different Melting Temperatures. Materials.

[6] Shuai C., Shuai C., Wu P., Yuan F., Feng P., Yang Y., Guo W., Fan X., Su T., Peng S. (2016). Characterization and Bioactivity Evaluation of (Polyetheretherketone/Polyglycolicacid)-Hydroyapatite Scaffolds for Tissue Regeneration. Materials.

[7] Fukuda H. (2015). Additive Manufacturing Technology for Orthopedic Implants in Advances in Metallic Biomaterials. Springer Ser. Biomater. Sci. Eng..

[8] Kim T.B., Yue S., Zhanga Z., Jones E., Jones J.R., Lee P.D. (2014). Additive manufactured porous titanium structures: Through-process quantification of pore and strut networks. J. Mater. Process. Technol..

[9] Zhao F., Li D., Jin Z. (2018). Preliminary Investigation of Poly-Ether-Ether-Ketone Based on Fused Deposition Modeling for Medical Applications. Materials.

[10] Zhong W., Li F., Zhang Z., Song L., Li Z. (2001). Short fiber reinforced composites for fused deposition modeling. Mater. Sci. Eng. A Struct..

[11] Karsli N.G., Aytac A. (2013). Tensile and thermomechanical properties of short carbon. Compos. Part B Eng..

[12] Shofner M.L., Lozano K., Rodriques-Macias F.J., Barrera E.V. (2013). Nanofiber-Reinforced Polymers Prepared by Fused Deposition Modeling. J. Appl. Polym. Sci..

[13] Fafenrot S., Grimmelsmann N., Wortmann M., Ehrmann A. (2017). Three-Dimensional (3D) Printing of Polymer-Metal Hybrid Materials by Fused Deposition Modeling. Materials.

[14] Makuzaki R., Masahito M., Namiki M., Jeong T.-K., Asahara H., Horiguchi K., Nakamura T., Todoroki A., Hirano Y. (2016). Three-dimensional printing of continuous-fiber composites by in-nozzle impregnation. Nat. Sci. Rep..

[15] Yang C., Tian X., Liu T., Cao Y., Li D. (2017). 3D printing for continuous fiber reinforced thermoplastic composites: Mechanism and performance. Rapid Prototyp. J..

[16] Melnikova R., Ehrmann A., Finsterbusch K. (2014). 3D printing of textile-based structures by Fused Deposition Modelling (FDM) with different polymer materials. IOP Conf. Ser. Mater. Sci. Eng..

[17] Gonzalez-Gutierrez J., Cano S., Schuschnigg S., Kukla C., Sapkota J., Holzer C. (2018). Additive Manufacturing of Metallic and Ceramic Components by the Material Extrusion of Highly-Filled Polymers: A Review and Future Perspectives. Materials.

[18] Popescu D., Zapciu A., Amza C., Baciu F., Marinescu R. (2018). FDM process parameters influence over the mechanical properties of polymer specimens: A review. Polym. Test..

[19] Ciacala G., Latteri A., Del Curto B., Lo Russo A., Recca G., Farè S. (2017). Engineering thermoplastics for additive manufacturing: A critical perspective with experimental evidence to support functional applications. J. Appl. Biomater. Funct. Mater..

[20] Gkartzou E., Koumoulos E.P., Charitidis C.A. (2017). Production and 3D printing processing of bio-based thermoplastic filament. Manuf. Rev..

[21] Vaezi M., Yang S. (2015). Extrusion-based additive manufacturing of PEEK for biomedical applications. Virtual Phys. Prototyp..

[22] Sabliov C.M., Fronczek E.C., Astete C.E., Khachaturyan M., Khachaturyan L., Leonardi C. (2009). Effects of Temperature and UV Light on Degradation of a-Tocopherol in Free and Dissolved Form. J. Am. Oil Chem. Soc..

[23] Bonan R., Asgar A. (2009). Biodegradable Stents-Where are we in 2009. US Cardiol..

[24] Mareci D., Bolat G., Izquierdo J., Crimu C., Munteanu C., Antoniac I., Souto R.M. (2016). Electrochemical characteristics of bioresorbable binary MgCa alloys in Ringer’s solution: Revealing the impact of local pH distributions during in-vitro dissolution. Mater. Sci. Eng. C Mater. Biol. Appl..

[25] Staiger M., Pietak A., Huadamai J., Dias G. (2006). Magnesium and its Alloys as Orthopedic Biomaterials: A review. Biomaterials.

[26] Bita A.I., Antoniac A., Cotrut C., Vasile E. (2016). In vitro Degradation and Corrosion Evaluation of Mg-Ca Alloys for Biomedical Applications. JOAM.

[27] Witte F., Fischer J., Nellesen J., Crostack J.H.A., Kaese V., Pisch A., Beckmann F., Windhagen H. (2006). In Vitro and In Vivo Corrosion Measurements of Magnesium Alloys. Biomaterials.

[28] Radha R., Sreekanth D. (2017). Insight of magnesium alloys and composites for orthopedic implant applications—A review. J. Magnes. Alloy..

[29] Antoniac I., Vranceanu M.D., Antoniac A. (2013). The influence of the magnesium powder used as reinforcement material on the properties of some collagen based composite biomaterials. JOAM.

[30] Erbel R., Di Mario C., Bartunek J., Bonnier J., de Bruyne B., Eberli F.R., Erne P., Haude M., Heublein B., Horrigan M. (2007). Temporary Scaffolding of Coronary Arteries with Bioabsorbable Magnesium Stents: A Prospective Non-Randomized Multi-Center Trial. Lancet.

[31] Xu L., Yamamoto A. (2012). Characteristics and cytocompatibility of biodegradable polymer film on magnesium by spin coating. Colloids Surf. B Biointerfaces.

[32] Gollwitzer H., Thomas P., Diehl P., Steinhauser E., Summer B., Barnstorf S., Gerdesmeyer L., Mittelmeier W., Stemberger A. (2005). Biomechanical and allergological characteristics of a biodegradable poly (d,l-lactic acid) coating for orthopaedic implants. J. Orthop. Res..

[33] Galliera E., Ragone V., Marazzi M.G., Selmin F., Banci L., Corsi Romanelli M.M. (2018). Vitamin E-stabilized UHMWPE: Biological response on human osteoblasts to wear debris. Clin. Chim. Acta.

[34] Kyomoto M., Moro T., Yamane S., Watanabe K., Hashimoto M., Takatori Y., Tanaka S., Ishihara K. (2014). Poly(2-methacryloyloxyethyl phosphorylcholine) grafting and vitamin E blending for high wear resistance and oxidative stability of orthopedic bearings. Biomaterials.

[35] Bracco P.E. (2011). Oral Vitamin E-stabilized UHMWPE for total joint implants: A review. Clin. Orthop. Relat. Res..

[36] Oral E., Godleski Beckos C., Malhi A.S., Muratoglu O.K. (2008). The effects of high dose irradiation on the cross-linking of vitamin E-blended ultrahigh molecular weight polyethylene. Biomaterials.

[37] Bladen C.L., Teramura S., Russell S.L., Fujiwara K., Fisher J., Ingham E. (2013). Analysis of wear, wear particles, and reduced inflammatory potential of vitamin E ultrahigh-molecular-weight polyethylene for use in total joint replacement. J. Biomed. Mater. Res. B Appl. Biomater..

[38] Ridley M.D., Jahan M.S. (2009). Effects of packaging environments on free radicals in gamma-irradiated UHMWPE resin powder blend with vitamin E. J. Biomed. Mater. Res. A.

[39] Renò F., Aina V., Gatti S., Cannas M. (2005). Effect of vitamin E addition to poly(d,l)-lactic acid on surface properties and osteoblast behaviour. Biomaterials.

[40] Renò F., Traina V., Cannas M. (2007). Haemocompatibility of vitamin-E-enriched poly(d,l-lactic acid). J. Biomater Sci. Polym. Ed..

[41] Misra S.K., Philip S.E., Chrzanowski W., Nazhat S.N., Roy I., Knowles J.C., Salih V., Boccaccini A.R. (2009). Incorporation of vitamin E in poly(3hydroxybutyrate)/Bioglass composite films: Effect on surface properties and cell attachment. J. R. Soc. Interfaces.

[42] Salawi A., Nazza S. (2018). The rheological and textural characterization of Soluplus®/Vitamin E composites. Int. J. Pharm..

